# Longitudinal associations between cardiovascular kidney metabolic syndrome and dementia in older adults

**DOI:** 10.1093/geroni/igaf122.667

**Published:** 2025-12-31

**Authors:** Benjumin Hsu

**Affiliations:** Harvard Medical School, Boston, Massachusetts, United States

## Abstract

**Aim:**

The American Heart Association (AHA) recently introduced the cardiovascular-kidney-metabolic (CKM) syndrome. The relationship between CKM syndrome and dementia in community-dwelling older men is unclear.

**Method:**

Community-dwelling men ages 70 years and older free of dementia from the CHAMP study (n = 1270) were assessed at baseline (2005-07). CKM syndrome was defined based on the Presidential Advisory from the AHA. Incidence dementia was identified over 17 years based on linked hospital records. The Cox proportional hazards model building for all analyses adjusted for relevant covariates.

**Results:**

In this longitudinal study of older men aged 76.8 ± 5.5 years, approximately 2% in stage 0, 2% in stage 1, 46% in stage 2, 19% in stage 3, and 32% were in stage 4. Our model demonstrated significant linear trend (p < 0.001) as the stages of CKM become more advanced in the risk of incident dementia. In the multivariable-adjusted model, participants with stage 4 CKM were statistically significantly associated with increased risk of developing dementia (HR: 1.76, 95%CI: 1.07-2.92) when compared to participants with no CKM or stage 0. Participants with stage 3 CKM (HR: 1.60, 95%CI: 0.96-2.66), stage 2 CKM (HR:1.33, 95%CI: 0.81-2.18) and stage 1 CKM (HR: 1.70, 95%CI: 0.87-3.31) may have increased risk of dementia when compared to stage 0 participants.

**Conclusion:**

Advanced stages of CKM syndrome is associated with dementia among community-dwelling older men. Older adults with CKM may need to be followed closely and managed in preventing dementia.

